# Targeted identification of TE insertions in a *Drosophila* genome through hemi-specific PCR

**DOI:** 10.1186/s13100-017-0092-1

**Published:** 2017-07-28

**Authors:** Shuo Zhang, Erin S. Kelleher

**Affiliations:** Department of Biology and Biochemistry, University of Houston, 3455 Cullen Blvd. Suite 342, Houston, TX 77204 USA

## Abstract

**Background:**

Transposable elements (TEs) are major components of eukaryotic genomes and drivers of genome evolution, producing intraspecific polymorphism and interspecific differences through mobilization and non-homologous recombination. TE insertion sites are often highly variable within species, creating a need for targeted genome re-sequencing (TGS) methods to identify TE insertion sites.

**Methods:**

We present a hemi-specific PCR approach for TGS of *P*-elements in *Drosophila* genomes on the Illumina platform. We also present a computational framework for identifying new insertions from TGS reads. Finally, we describe a new method for estimating the frequency of TE insertions from WGS data, which is based precise insertion sites provided by TGS annotations.

**Results:**

By comparing our results to TE annotations based on whole genome re-sequencing (WGS) data for the same *Drosophila* *melanogaster* strain, we demonstrate that TGS is powerful for identifying true insertions, even in repeat-rich heterochromatic regions. We also demonstrate that TGS offers enhanced annotation of precise insertion sites, which facilitates estimation of TE insertion frequency.

**Conclusions:**

TGS by hemi-specific PCR is a powerful approach for identifying TE insertions of particular TE families in species with a high-quality reference genome, at greatly reduced cost as compared to WGS. It may therefore be ideal for population genomic studies of particular TE families. Additionally, TGS and WGS can be used as complementary approaches, with TGS annotations identifying more annotated insertions with greater precision for a target TE family, and WGS data allowing for estimates of TE insertion frequencies, and a broader picture of the location of non-target TEs across the genome.

**Electronic supplementary material:**

The online version of this article (doi:10.1186/s13100-017-0092-1) contains supplementary material, which is available to authorized users.

## Background

Transposable elements (TEs) are mobile genetic entities that are major contributors to the evolution of eukaryotic genomes. TE proliferation can drive dramatic changes in genome size [1–4] and gene regulation [5–8]. Additionally, ectopic recombination between TE insertions produces structural rearrangements within and between chromosomes [9–13]. Finally, transposition into novel genomic sites produces abundant intraspecific variation in the presence and absence of individual TE insertions [14–16].

Despite their contribution to genetic variation, population genomic studies of TEs remain challenging. Like all repetitive elements, TEs are inherently problematic to assign to particular genomic locations. Furthermore, TEs are often found in heterochromatic regions, such that the genomic sequences that surround them may also be repetitive. Finally, TE insertions are often polymorphic within samples used for genome re-sequencing, meaning they are supported by few sequencing reads, and discerning between false positives and rare insertions can prove difficult [17–20].

Whole genome re-sequencing (WGS) is often employed to provide a comprehensive picture of genetic variation, including the presence and absence of TE insertions. Numerous methodologies have been developed for annotation of polymorphic TE insertions from WGS [17–23]. However, WGS of a large population genomic sample remains expensive, and may be unnecessary for studies that focus on one or a few active TE families. Additionally because WGS provides variable sequence coverage across the genome, and the power to annotated particular TE insertions may be limited by stochastic low read-depth. Read depth may be critical for identification of a unique TE insertion site, particularly in heterochromatic repeat-rich regions that contain limited unique sequence.

Targeted genomic re-sequencing (TGS) of TE insertions allows for vastly increased sequencing depth at TE insertion sites in smaller sequencing libraries as compared to WGS [24–26]. TGS therefore offers combined potential for more robust identification of TE insertions that are rare or occur in repetitive regions, at a reduced sequencing cost. Here, we adapt a hemi-specific PCR approach for TGS of TE insertions on the Illumina platform [24] to *Drosophila* genomes. We further present a computational method for identification of precise TE insertion sites from TGS data. Although our approach is adaptable to any TE or genome, we piloted it by re-sequencing insertions of *P-*elements, DNA transposons that recently invaded the *D. melanogaster* genome and are highly polymorphic among strains [27–32]. To evaluate our approach, we compared our results to two TE annotation sets based on WGS data for the same strain [18, 19, 33].

We demonstrate that TGS by hemi-specific PCR is a powerful method for identification of polymorphic *P-*element TE insertions in *Drosophila*, identifying almost all known insertions (~94%), while also uncovering previously un-annotated insertions in repetitive genomic regions. False-positives in TGS data were easily differentiated from true insertions based on read support. We further demonstrate that TGS allows for identification of precise insertion sites for all annotated TEs, as compared to WGS, where the absence of reads spanning the TE insertion breakpoint often limits the resolution of the annotations to a genomic window. Finally, we describe a new method for estimating the polymorphic frequency of individual TE insertions from WGS data, which takes advantage of precise insertion sites provided by TGS. Overall, our results suggest that TGS based on hemi-specific PCR may be a more powerful and precise method for annotation of polymorphic TE insertions than WGS for the study of particular TE families, such as the *P*-element. However, the two approaches are complementary, and together provide the most complete picture of TE location and frequency.

## Results

### Hemi-specific PCR amplifies abundant *P*-element insertions

*P-*elements are absent from the *D. melanogaster* reference genome (*y* ^*1*^ *; cn* ^*1*^ *bw* ^*1*^ *sp* ^*1*^) [34], but are ubiquitous among recently collected wild-type genomes [18, 19]. We therefore chose to pilot our approach by examining *P-*elements in the wild-derived strain RAL-492, which was collected from Raleigh NC in 2003 [35]. Illumina paired-end whole-genome sequencing data was previously published for RAL-492, and genomic *P-*elements were previously annotated by the TEMP (33 insertions [18]) and TIDAL (29 insertions [19]) TE annotation packages.

**Fig. 1:**
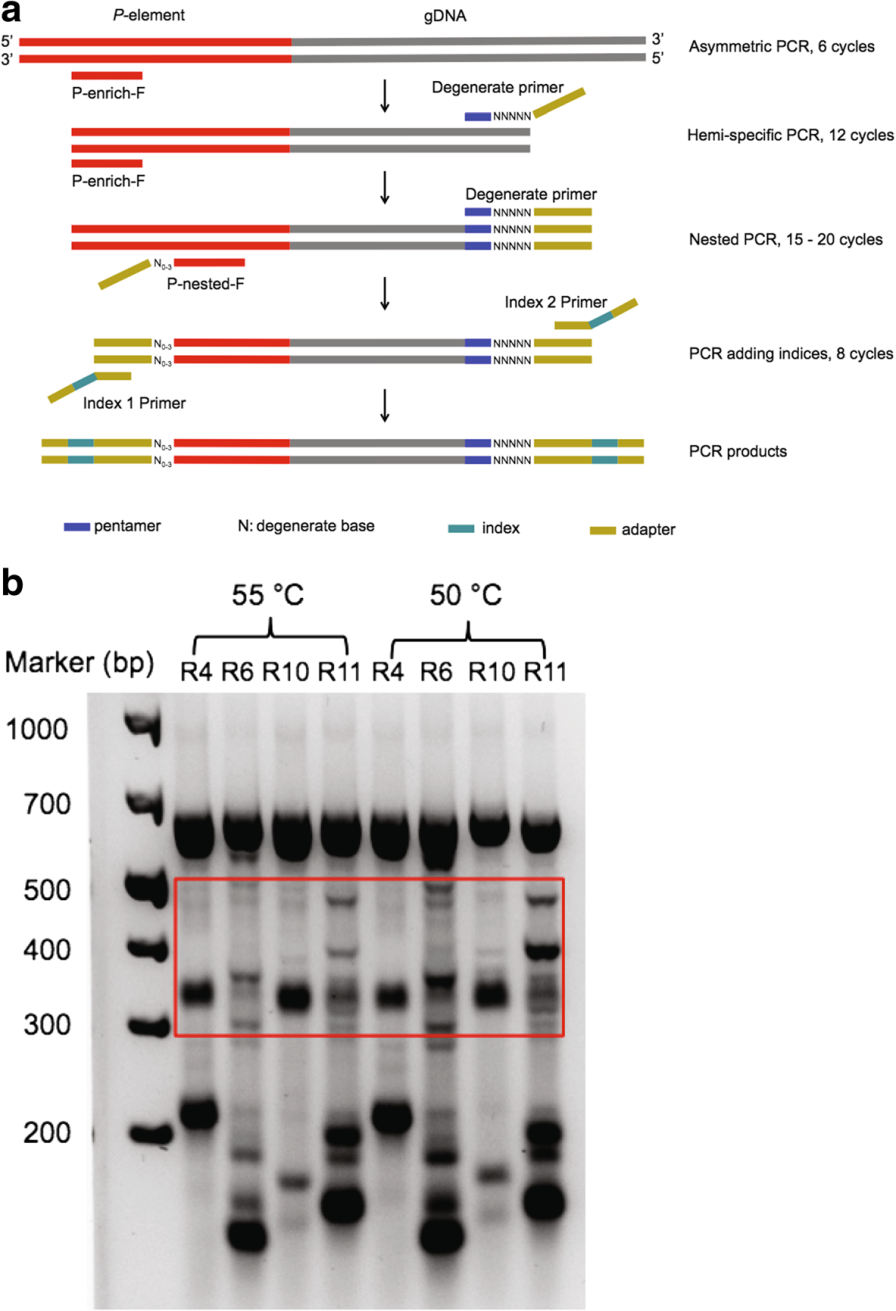
Hemi-specific PCR of *P-*element insertions. **a** Sequencing libraries were generated by nested hemi-specific PCR. First, asymmetric PCR enriches for *P*-element 3’ends using a *P*-element specific primer (P-enrich-F) that aligns to *P*-element from position 2752 to 2774 (out of 2907 total nucleotides). Next, a degenerate reverse primer is added recognize and amplify unknown sequences that are adjacent to *P*-element 3′ ends. Third, nested PCR with the P-nested-F primer cocktail (positions 2856 to 2877) and the degenerate reverse primer enhances PCR specificity for *P-*elements and produces amplicons with 5′ end read complexity, which is required for Illumina sequencing. Last, DNA fragments are amplified with indexing primers to allow for multiplexing. The resulting amplicons consist of adapters at each end, a *P*-element 3′ end and its adjacent genomic sequences. **b** PCR products from nested PCR with four degenerate primers (R4, R6, R10 and R11) are shown for two different annealing temperatures

To amplify *P-*element insertions and adjacent sequence the from RAL-492 genome [35], we employed a hemi-specific PCR approach, using a forward primer specific to a region at the 3′ end of *P*-elements that is required for transposition [36], and a series of 15 degenerate reverse primers (Fig. 1a). Each degenerate reverse primers contains a different common pentamer in the *D. melanogaster* genome followed by 5 four-fold degenerate nucleotides (N bases), allowing it to recognize a diversity of chromosomal sites (Additional file 1: Table S4). To determine the optimal annealing temperature for hemi-specific PCR, and verify that our approach would amplify a range of DNA fragments corresponding to multiple *P-*element insertions, we examined the size distribution of amplicons for 4 degenerate primers at two different annealing temperatures (55 °C and 50 °C, Fig. 1b). Although a diversity of fragment sizes were observed for both annealing temperatures, the range was broader and more evenly distributed among amplicons at 50 °C. We therefore separately conducted hemi-specific PCR for 15 degenerate primers at the annealing temperature of 50 °C to generate our sequencing libraries.

We sequenced 0.43–1.31 million read pairs for each of 15 degenerate primers (Additional file 2: Table S1). >93% of read pairs for all 15 degenerate primers contained 3′ *P*-element sequences, indicating our PCR conditions were highly specific (Additional file 2: Table S1). After trimming *P-*element sequence and low-quality ends, we aligned read pairs to release six of the *D. melanogaster* genome (dm6) [34], and the Telomere Associated Satellites of the *X*-chromosome (*X*-TAS) [37]. Although *X*-TAS is absent from the genome of the dm6 reference strain (*y* ^*1*^ *; cn* ^*1*^ *bw* ^*1*^ *sp* ^*1*^) [34], these subtelomeric satellites are common among wild-derived genomes and often contain *P-*elements [38–41]. Depending on the degenerate primer, 80.8 – 98.0% of read pairs were aligned to the reference, with 20.8 – 97.3% of read pairs aligning to the reference in unique genomic location (Additional file 2: Table S1). Therefore, there is variation among the degenerate primers in the degree to which the insertions they amplify are surrounded by unique genome sequence.

**Fig. 2:**
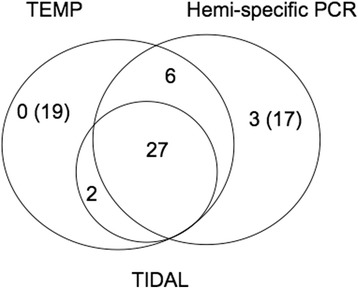
The number of *P*-element insertions found by Hemi-specific PCR, TEMP and TIDAL. The number of *P*-element insertions is indicated in each subset. The number in parentheses indicates the number of known or potential false positives

To identify *P-*element insertions from our sequencing reads, we first considered read pairs that could be uniquely mapped to the reference genome (see Methods). In total, 53 independent *P*-element insertion sites were suggested in the RAL-492 genome, based on the unique and concordant alignment of >20 *P-*element derived read pairs to the reference for each insertion (Additional file 3: Table S2). Of these 53 insertions, 27 had previously been identified from WGS data by both TIDAL [18] and TEMP packages [19], and an additional 6 had been identified by TEMP only (Fig. 2). By contrast, only 2 insertions found by TIDAL and TEMP were not detected by hemi-specific PCR. Hemi-specific PCR therefore identified almost all high-confidence *P-*element insertions detected in whole genome re-sequencing data while also suggesting up to 20 previously unknown insertions.

To determine why hemi-specific PCR may fail to detect a small number of insertions, we examined the insertion sites of the two *P-*elements annotated by both TIDAL and TEMP but not hemi-specific PCR. We discovered that in both cases, the annotated insertions were two tail-to-tail *P-*element insertions, meaning that amplification from the 3′ end of one element would produce sequence from the 3′ end of the adjacent element, rather than genomic sequence corresponding to the insertion site. False negatives could therefore be avoided with this method in the future by placing *P-*element specific primers at both the 5′ and 3′ ends of the element.

We also did not detect 19 *P*-element insertions that were found only by TEMP (Fig. 2). Notably, these insertions were excluded from the published TEMP annotations because they were note estimated to occur at more than 80% frequency in any inbred line, including RAL-492 [18]. If these insertions are true positives that are segregating at a low frequency in RAL-492 (Additional file 4: Figure S1A), they may not have been represented in the sample of genomic DNA that we used for Illumina library prep. Alternatively, these insertions may be false positives, as they are supported by fewer read-pairs in whole genome re-sequencing data than those that were also identified by TIDAL, hemi-specific PCR, or both (Additional file 4: Figure S1B). Indeed, we attempted to amplify one of these insertions using standard PCR and were unable to do so (Additional file 5: Table S3).

### Validation of novel insertions and identification of false positives

To validate the 20 candidate novel *P*-element insertions identified by hemi-specific PCR we performed site-specific PCR. Among the *P*-element insertions found only by hemi-specific PCR (Fig. 2), 3 insertions (chr2L:20,917,521, chrX_TAS:4894 and chrY:768,808) could be amplified from RAL-492 genomic DNA (Additional file 5: Table S3). Insertions at chrX_TAS:4894 and chrY:768,808 appear to be fixed in the RAL-492 strain, and we were able to identify read pairs (15 for chrX_TAS:4894 and 18 for chrY:768,808) in the previous WGS data that support these two insertions. However, because these insertions are located in repetitive genomic regions, there were no read pairs in the WGS data that uniquely aligned to either insertion site, preventing their detection by TEMP and TIDAL. The read depth provided by TGS therefore offers greater power to identify TE insertions in heterochromatic regions. The third insertion, chr2L:20,917,521 is polymorphic, as indicated by the presence of PCR amplicons corresponding to both inserted and un-inserted chromosomes (Additional file 6: Figure S2). There were no read pairs supporting this polymorphic insertion in the previous WGS data, perhaps because the inserted chromosome was not sampled among individuals used for the sequencing library.

**Fig. 3:**
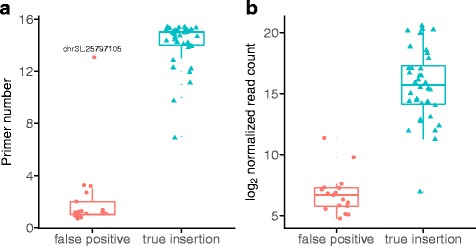
Read and primer support for true insertions and false positives detected by hemi-specific PCR. False-positives were detected by hemi-specific PCR but could not be validated by insertion-specific PCR or whole genome re-sequencing data, whereas true insertions were verified by one or both of these methods. **a** True insertions are sampled more sequencing libraries generated using different degenerate primers for hemi-specific PCR (Welch’s *t*_22_ = 15.56, *P* = 2.91 × 10^−13^). **b** True insertions are supported by larger number of uniquely mapping read pairs in hemi-specific PCR libraries (Welch’s *t*_50_ = 13.78, *P* < 2.2 × 10^−16^). The number of read pairs was normalized to reads per million based on total sequenced reads from each degenerate primer

We could not validate the remaining 17 insertions that were uniquely identified by hemi-specific PCR, either through insertion-specific PCR or from previous whole-genome sequencing data (Additional file 5: Table S3). We therefore believe these are false positives that result from PCR artifacts that occur during library prep. Fortunately, false positives are easily distinguished from true insertions by the low abundance of supporting reads among our sequencing libraries and their presence in sequencing libraries from only a few degenerate primers (Fig. 3). If we require at least 100 read pairs and 4 degenerate primers to define a *P*-element insertion, we are able to exclude all but one of the false positives. Excluding false-positives, we detected 36 *P*-element insertions in the RAL-492 genome, three of which were previously unknown (Additional file 3: Table S2).

### Sequence similarity to true insertion sites may produce false positives

There is one outlier among the false positives: an insertion at chr3L:25,797,105 (Fig. 3a) that is supported by 1478 read pairs and 13 degenerate primers. Notably, we found the sequence around this insertion site was 94% similar across 446 bp to sequence at a true insertion site (chr3L:26,023,661). Therefore, some false positives may occur due to nucleotide substitutions introduced during PCR and sequencing, which cause a subset of reads derived from a true insertion to align better to highly similar sequences elsewhere in the genome. Consistent with this, the reads supporting the false positive were 0.17% as abundant in our data as compared to reads supporting the true insertion (Additional file 3: Table S2), which is similar what is expected based on the per-site mutation rate for Taq DNA polymerase (0.003%) [42] and the Illumina MiSeq platform (0.8%) [43]. Furthermore, reads supporting the true insertion site were separated by fewer mutations from the reference genome (mean 2.2 mutations per 100 bp) as compared to reads supporting the false positive insertion (mean 6.7 mutations per 100 bp).

To determine whether sequence identity might explain other potential false positives we observed in our data, we compared 0.8 Kb of the genomic region surrounding all insertion sites to each other via BLAST [44]. We found that the genomic sequence at two potential false positives chr3L:26,834,988 and chrUn_CP007074v1:15,794 exhibited significant sequence similarity to the PCR-verified insertion chrX_TAS:4894 (87% across 83 bp for chr3L:26,834,988; 84% identity across 93 bp for chrUn_CP007074v1:15,794). In both cases, reads supporting the potential false-positive insertions were <1% as abundant as reads supporting the true positive (Additional file 3: Table S2).

### The majority of sequencing reads are explained by annotated insertions

For some degenerate primers, >50% of read pairs aligned to the reference genome in multiple location (i.e. multiply mapping Additional file 2: Table S1). These read pairs might be derived from one of the 36 insertions that were annotated from unique alignments. Alternatively, they may indicate the presence of false negatives, which could not be annotated due to an absence of uniquely mapping reads. To differentiate between these alternatives, we constructed a putative contig for each of the 36 *P-*element insertions, which was comprised of the full-length *P-*element consensus flanked by 500 nucleotides of adjacent genomic sequence (see Methods). Multiply mapping reads that support annotated insertions were then identified based on their alignment to the 36 putative insertion contigs.

For all but one of the degenerate primers, >95% of multiply mapping reads could be aligned to at least one of the 36 putative insertion contigs (Additional file 2: Table S1). Furthermore, most multiply mapping reads were aligned to insertions in repetitive genomic regions, such as chrX_TAS:4894. Therefore, with the exception of the tail-to-tail elements, our analysis pipeline likely detects most or all of the *P-*elements present in hemi-specific Illumina libraries.

### Improved insertion site identification and frequency estimation

**Fig. 4:**
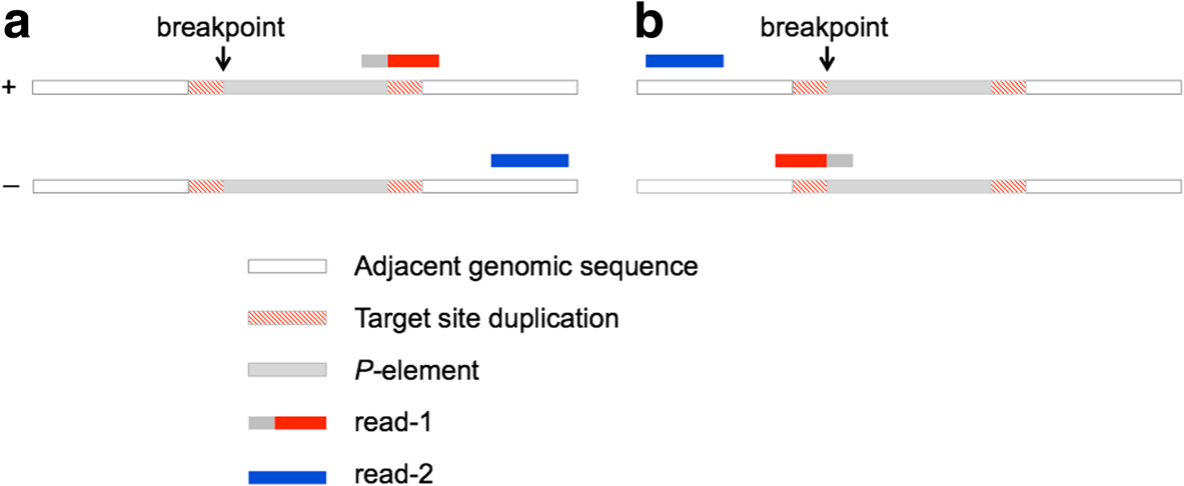
Insertion Site Identification and Putative Insertion Contig Structure. Read-1 of each pair generated by hemi-specific PCR is a split read that contains both *P*-element and adjacent genomic sequence. Breakpoints are determined based on the alignment of read-1 (red) to the plus (**a**) or minus genomic strand (**b**). Contigs are constructed through insertion of the P-element consensus at the insertion site, which is flanked by an 8 bp target site duplication on either side

Read-pairs generated by hemi-specific PCR include at least one “split-read” which is comprised of both TE and adjacent genomic sequences. Split reads are invaluable for TE annotation, because they allow for precise identification of the breakpoint that characterizes each insertion (Fig. 4), but are often absent from annotations based on WGS data due to lower read depth at individual insertion sites. For example, although the precise insertion site of all 36 insertions detected in the RAL-492 genome by hemi-specific PCR were identified, 5 of these insertion sites were absent from TEMP annotations based on WGS data, due to a lack of split reads [18]. An additional 5 insertions had slightly different insertion sites inferred by hemi-specific and WGS, suggesting potential inaccuracy in annotation of the insertion site.

Precision and accuracy of insertions site annotation could be of particular value in facilitating the estimation of polymorphic TE insertion frequencies from WGS data. TE annotation packages such as TEMP and TIDAL estimate the frequency of an individual TE insertion among sequenced genomes as the proportion of read pairs aligning to the insertion site that support the insertion allele. However, because precise insertion sites are not always known, reads supporting each chromosome cannot be identified by concurrent alignment to the reference genome and a putative insertion allele. Rather, reads are aligned to the reference genome only, and read-pairs supporting the insertion allele are identified by a minimal number of nucleotides (7 nt for TEMP and 22 nt for TIDAL) that align to the TE consensus. Such an approach likely underestimates the number of reads supporting the insertion chromosome by excluding read-pairs that include very little TE sequence.

Taking advantage of the precise breakpoints that are provided by hemi-specific PCR, we developed a new method for estimating the frequency of polymorphic TE insertions in WGS data. Unfortunately, the frequency of the insertion allele cannot be estimated from TGS data, because reads supported the reference allele (lacking a TE insertion) are not represented in the sequencing library. We aligned WGS reads concurrently to the reference genome as well as putative contigs for each of the 36 annotated insertions. We then estimated the frequency of each *P-*element insertion based on the number of read-pairs in WGS data that exhibit a significantly better alignment to the putative insertion contig than to the corresponding window in the reference genome.

**Fig. 5:**
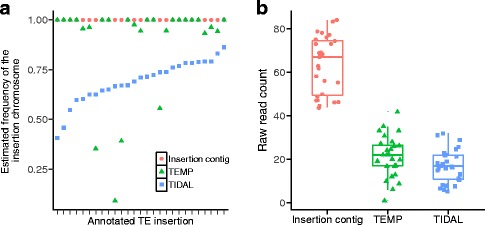
Estimation of TE insertion frequency. **a** Estimated frequencies for 27 TE insertions in RAL-492 generated by TEMP, TIDAL, and our concurrent alignment approach (insertion contig). All three frequency estimates are based on previously published WGS data from RAL-492 [35]. **b** The number of WGS read pairs supporting each *P*-element insertion identified by TIDAL, TEMP and concurrent alignment (contig)

Based on this approach, we estimate that 97.2% (35 out of 36) of the *P-*element insertions identified by both TEMP and hemi-specific PCR are completely fixed in RAL-492, as expected in a highly inbred line. By contrast, using the same WGS data as we employed, TIDAL and TEMP estimated that many insertions remained polymorphic after inbreeding (Fig. 5a). Specifically, for the 27 insertions found by TEMP, TIDAL and hemi-specific PCR (Fig. 2), the median frequency estimated from concurrent alignment to the reference and putative insertion contig was 0.31 higher than the TIDAL estimate (*P* < 1 × 10^−6^, based on 10^6^ permutations of the observed data) and 0.11 higher than the TEMP estimate (*P* = 5.1 × 10^−4^, based on 10^6^ permutations of the observed data). The higher estimated TE insertion frequencies generated by concurrent mapping resulted from a larger number of identified read pairs that support the insertion chromosome, as compared to the TIDAL and TEMP approaches (Fig. 5b; linear contrast *F*_*1,54*_ = 564.54, *P* < 2 × 10^−16^). Furthermore, TIDAL generated the lowest estimated frequencies and the fewest reads supporting the inserted chromosome, which is consistent with the most stringent requirements for identification of reads supporting the insertion (22 nt overlap with the consensus).

For six insertions, we validated that the insertion was fixed in our RAL-492 sample by performing PCR with primers on either side of the insertion site, such that both the insertion allele and the reference (un-inserted) allele would amplify if present. Only the insertion allele amplified, suggesting that the reference allele was absent. Collectively, our observations suggest a systematic bias towards low TE insertion frequency estimates when reads are not aligned to a putative insertion contig that is defined by precise breakpoints.

## Discussion

Our results validate hemi-specific PCR as a powerful method for TGS of particular TE families. Of 38 true insertions in the RAL-492 genome, which were either independently validated by site-specific PCR (Additional file 5: Table S3), or were found in multiple annotation sets (Additional file 3: Table S2), 36 could be identified from sequencing reads generated by hemi-specific PCR. By contrast, TEMP detected 35 true insertions [18] while TIDAL detected 29 [19] (Fig. 2). Hemi-specific PCR therefore exhibited marginally to significantly improved power to detect true insertions when compared to previous analyses of WGS data, based on ~50% fewer sequencing reads (Additional file 2: Table S1) [35]. Furthermore, given that all but one true insertion was supported by >1000 uniquely mapping reads in our data (Additional file 3: Table S2), hemi-specific PCR libraries could be highly multiplexed while still retaining power to discover the vast majority of insertions. Importantly, we were able to avoid almost all false positives by excluding insertions that were supported by few reads or degenerate primers (Fig. 3), revealing that the enhanced power of TGS for genome annotation does not come at the expense of accuracy. By contrast, TEMP annotation of WGS data detected almost all true insertions but also exhibited a high false positive rate, while TIDAL avoided false positives but missed many true insertions (Fig. 3, Additional file 5: Table S3).

Annotating TE insertions in heterochromatic regions based on WGS data remains challenging, as individual insertions are often supported by only few read pairs, which may not yield a unique alignment in repeat rich sequence. Annotation of polymorphic TE insertions in heterochromatic regions is of particular interest due to the known role of heterochromatic piRNA clusters in regulating germline TE activity in both mammals and insects [45, 46]. TGS by hemi-specific PCR offered improved annotation in heterochromatic regions, as two of the three previously un-annotated insertions we discovered here were in heterochromatin. Indeed, one of the previously unknown insertions we annotated is in the *X*-*TAS*, a prolific piRNA cluster [45] that plays an important role in *P-*element regulation [39–41, 47, 48]. TGS by hemi-specific PCR may therefore provide an opportunity to examine polymorphic TE insertions that determine differences in TE regulation [49].

Our TGS and analysis method, based on hemi-specific PCR, also provided precise insertions sites for all annotated TEs, which are often lacking from annotations based on WGS data. Precise insertion sites provide more information about the potential functional impact of a TE insertion. Additionally, as we demonstrated, they allow for more accurate estimates of the polymorphic frequency of TE insertions from WGS data. Estimating TE insertion site frequencies is critical for examining the selective forces that act on TE insertions [15, 17, 50]. They are also important to consider when evaluating associations between particular TE insertions and phenotypes of interest in genome-wide association studies.

## Conclusion

Our results indicate that hemi-specific PCR offers an attractive alternative approach to WGS for identification of polymorphic TE insertions of particular TE families in *Drosophila* genomes*.* As expected for a targeted approach focused on a single TE family, TGS was more powerful for annotating true positive *P-*element insertions than WGS, and also offered enhanced precision and accuracy in determining the exact location of those insertions. Furthermore, this performance was achieved at a lower read depth and therefore reduced sequencing cost.

TGS is easily adapted to other host genomes or TE families through development of new nested and degenerate primer sets. Indeed our method is modeled after that of Ewing and Kazazian [24], which curated LINE-1 elements in human genomes. Additionally, TGS could be expanded to identify polymorphic insertions for many TE families in the same library by incorporating multiple nested primer pairs. Such an approach would be invaluable for population genomic studies that focus on the dynamics of particular active TE families.

## Methods

### Genomic DNA samples

RAL-492 and RAL-802 strains were obtained from the Bloomington *Drosophila* Stock Center. Genomic DNA was extracted using the Qiagen DNeasy Blood and Tissue kit.

### Primer design

Our library preparation method is modeled after the approach described by Ewing and Kazazian [24], which amplifies LINE-1 elements and adjacent genomic sequences in human genomes (Fig. 1a). By combining nested forward primers that are specific to 3′ end of *P*-element with degenerate reverse primers, we preferentially amplified *P-*elements and their adjacent genomic sequences. The first *P-*element specific primer (P-enrich-F) enriches 3′ *P-*element ends, while the second (P*-*nested-F) contains Illumina nextera adapter sequences to allow for sequencing of amplicons. The nested forward primers used for PCR bind to sequences that are required for *P-*element mobilization, and therefore are expected to a conserved among genomic *P-*elements [36]. In addition, the forward nested primer was an equimolar cocktail of four different primers, which are complementary to the same stretch of the *P*-element 3′ end (position 2856 to 2877), but have spacers of 0–3 “N” nucleotides from the Illumina adaptor sequence (Fig. 1a). The spacers ensure sequence complexity at the start of the sequencing read, which is critical to the success of the sequencing reaction.

To design degenerate reverse primers for hemi-specific PCR, we first identified common pentamers in the *D. melanogaster* genome with jellyfish [51]. We selected a set of 15 pentamers that are common, but also diverse in their sequence composition, to maximize the breadth of genomic sequences that could be recognized by the degenerate primers. Each degenerate primer was comprised of an Illumina adapter for nextera sequencing, followed by 5 degenerate nucleotides, followed by a common pentamer from 5′ to 3′. Primers used in library construction are listed in Additional file 1: Table S4.

### Library construction by hemi-specific PCR

The first 6 cycles of PCR were asymmetric, and enriched for the 3′ end of *P*-elements. The PCR was conducted in a 46 μL reaction volume with 10 μL of 5X GoTaq Flexi Buffer (Promega), 6 μL of 25 mM MgCl_2_, 2 μL of 20 μM P-enrich-F primer, 0.5 μL of 100% DMSO, 0.5 μL of Flexi GoTaq, 1 μL of 10 mM dNTPs, and ~500 ng template DNA. The PCR conditions were 2:30 min at 95 °C, followed by 6 cycles of 30 s at 95 °C, 1 min at 62 °C and 2 min at 72 °C.

The second PCR was hemi-specific, and allowed for 12 cycles of amplification of *P-*element 3′ ends and adjacent genomic sequences. 4 μL of each degenerate primer (5 μM) was added to a separate asymmetric PCR reaction mix. The reaction conditions were 2 min at 95 °C, followed by 12 cycles of 30 s at 95, 30 s at 50 °C and 2 min at 72 °C, followed by 10 min at 72 °C. The PCR product was purified using the QIAquick PCR Purification Kit (Qiagen), yielding 20 μL DNA.

The third PCR (15–20 cycles) was nested, and provides enhanced specificity for *P-*element targets. Purified PCR products from PCRs 1 and 2 were used as templates, and amplification was targeted by an Illumina-tagged forward nested *P-*element primer, and the same degenerate reverse primer employed PCR 2. The PCR was conducted in 50 μL reaction volume with 10 μL of 5X GoTaq Flexi Buffer, 6 μL of 25 mM MgCl_2_, 4 μL of 5 μM equimolar forward primer, 4 μL of degenerate primer, 0.5 μL of 100% DMSO, 0.5 μL of Flexi GoTaq, 1 μL of 10 mM dNTPs, and 10 μL template DNA from last step. The PCR condition is: 2 min at 95 °C, followed by 15–20 cycles of 30 s at 95 °C, 30 s at 55 °C and 30 s at 72 °C, followed by 10 min at 72 °C. For degenerate primers R4, R6, R8, R9, R11, R12, R13, R15, PCR 3 was performed for 15 cycles. Because the remaining degenerate primers yielded weak bands or no bands after 15 cycles, we increased the number of cycles to 20 for these primers. For all 15 libraries, 300–500 bp PCR products were isolated from agarose gels and purified using the QIAquick Gel Extraction Kit (Qiagen), and 22. 5 μL purified DNA were eluted.

The fourth PCR (8 cycles) incorporated indices for multiplexing on the Illumina platform using the Illumina Nextera XT Index Kit. The PCR was conducted in a 50 μL reaction volume with 10 μL of 5X GoTaq Flexi Buffer, 6 μL of 25 mM MgCl_2_, 5 μL of index 1, 5 μL of index 2, 0.5 μL of Flexi GoTaq, 1 μL of 10 mM dNTPs, and 22.5 μL template DNA from last step. The PCR conditions were: 3 min at 95 °C, followed by 8 cycles of 30 s at 95 °C, 30 s at 55 °C and 30 s at 72 °C, followed by 5 min at 72 °C. PCR products between 300 and 500 bp were isolated from an agarose gel, and purified using the QIAquick Gel Extraction Kit. The resulting sequencing libraries were paired-end sequenced (2 × 150 nt reads) on the MiSeq platform by the Weill Cornell Epigenomics Core. Sequencing libraries are available in the NCBI sequence read archive (SRR5712353 to SRR5712367).

### Identification of *P*-element-derived read-pairs and alignment to the reference genome

Based on the placement of the P-nested-F primer, read-1 from every read pair should begin with 52 nt at the 3′ terminus of the *P*-element consensus (Fig. 1a). The first 22 nt are included in the P-nested-F primer, while the remaining 30 will occur only in amplicons that arise from true *P-*element 3′ ends. We therefore locally aligned all read-1 sequences to the full-length *P*-element consensus sequence [52] using bowtie2 (v2.1.0) [53] and selected read pairs where the alignment of read-1 to 3′ end of *P*-element was longer than 20 nt using a custom Perl script (1 mismatch and 1 gap allowed; Additional files 7 and 8). Any remaining Illumina sequencing adapters and *P*-element sequences, as well as low-quality ends, were removed from our selected read pairs using cutadapt (v1.9.1) [54]. The *P*-element derived and trimmed read pairs were used for all down-stream analyses (Additional file 2: Table S1).

### Annotation of *P*-element insertions based on uniquely mapping read pairs

To pinpoint *P*-element insertions in the RAL-492 genome, read pairs were globally aligned to dm6 as well as *X*-TAS using bowtie2 with default options. The results of alignments to the reference genome are reported in Additional file 2: Table S1. For read pairs that concordantly (i.e. aligned with expected orientation and the distance between mates is within 500 bp) and uniquely aligned to the reference genome, we determined the breakpoints of *P*-element insertions based on the reported alignments using a custom Perl script (Additional files 7, 9 and 10). As *P*-element transposition will generate 8-bp target site duplications [55], we defined breakpoints as the 3′ end of the 8-bp target site duplication on the plus genomic strand. If the *P*-element insertion is in the same orientation as the plus genomic strand, the breakpoint is equal to the location where left-most nucleotide was aligned in read-1 plus 7 bp (Fig. 4a). In contrast, the breakpoint is equal to location where the right-most nucleotide was aligned in read-1 if the inserted *P*-element is in the same orientation as the minus genomic strand (Fig. 4b). We required 20 concordant, uniquely mapping read pairs to annotate a single insertion. *P*-element insertions found by uniquely mapping read pairs were reported in Additional file 2: Table S1.

### Determining the number of *P*-element reads that arise from annotated insertions

To determine how many multiply mapping reads could be derived from one of the 36 insertions we annotated based on unique and concordant alignment to the reference genome, we aligned multiply mapping reads to putative insertion contigs that we generated for each annotated insertion. Each of the ~300–500 bp PCR products that were sequenced contain 52 bp of *P-*element sequence and 77 bp of Illumina adapter sequence, with the remaining sequence (up to ~371 bp) deriving from the genomic region adjacent to each insertion. We therefore constructed putative insertion contigs that contained the *P*-element consensus and 500 bp adjacent genomic sequences at 5′ and 3′ end, including the inferred 8 bp target site duplication (Fig. 4). Multiply-mapping read pairs were aligned to the putative insertion contigs using bowtie2, allowing for up to 5 mismatches and 2 gaps. The number of multiply mapping read pairs that could be aligned to at least one annotated insertion are listed in Additional file 2: Table S1.

### Estimating the frequency of individual insertions from whole genome sequencing paired-end data

To estimate frequency of each annotated TE insertion, we used previously published whole genome re-sequencing data for RAL-492 [35] to compare the abundance of read pairs supporting the insertion allele and reference genome. Read pairs were globally aligned to a hybrid assembly that combined the putative insertion contig for each of our insertions, as well as the dm6 assembly, using bowtie2. Only alignments with a mapping quality score (MAPQ) greater than 10, indicating high confidence that they are the correct alignment for a particular read-pair, were retained. A read pair was considered to support the insertion if it aligned to the putative insertion contig and its alignment spanned the breakpoint. Similarly, a read pair was considered to support the reference genome if it aligned to dm6 and the alignment spanned the breakpoint. The frequency of the TE insertion was estimated the proportion of the number of read pairs supporting the insertion out of total number of read pairs supporting either the inserted or un-inserted chromosomes.

### Site-specific PCR

To verify the existence of *P*-element insertions found by hemi-specific PCR and other approaches, we designed two different types of PCR assays. Insertion site assays combined forward and reverse primers on either side of each insertion site, such that potential PCR products would include both the reference and the insertion allele. Breakpoint-specific assays combined a *P*-element specific primer and a primer in the adjacent genomic sequence, and were specific to the insertion allele. PCR products were Sanger sequenced to further verify the presence or absence of *P*-element insertions. The primers for each insertion site we examined, as well as the PCR and sequencing results, are summarized in Additional file 4: Table S3.

With the exception on the *X-TAS* insertion*,* primers for site-specific PCR amplify a unique location in the reference genome. Even repetitive genomic regions often carry distinct combinations of adjacent repeats that allow for site-specific PCR. For the *X-TAS* insertion, we used a break point specific assay combining a primer anneals to a satellite sequence that is unique to *X-TAS* array [56] with a *P-*element specific primer. A positive result is diagnostic of a *P-*element insertion in a particular orientation in the *X-TAS* locus.

## Additional files

Additional file 1: Table S4. Primers used in library construction. (XLSX 47 kb)

Additional file 2: Table S1. The summary of paired-end reads. (XLSX 56 kb)

Additional file 3: Table S2. Insertions found by concordantly and uniquely mapping read pairs. (XLSX 42 kb)

Additional file 4: Figure S1. The *P*-element insertions found only by TEMP have a low frequency (A) and are supported by few reads (B). *P*-element insertion detected by TEMP (two tail-to-tail insertions are excluded) are divided into two groups: the “TEMP only” group which contains insertions found only by TEMP, and the “All” group which contains insertions found by TEMP, TIDAL and hemi-specific PCR. Frequency (called penetrance by TEMP) was estimated from whole genome sequencing of the RAL-492 line. TE read count indicates the number of paired-end reads that support the inserted chromosome. Insertions annotated only by TEMP have a significantly lower estimated polymorphic frequencies than insertions also detected by TIDAL and hemi-specific PCR (Welch’s *t*_41_ = 14.44, *P* < 2.2 × 10^−16^). In addition, the average number of reads supporting *P*-element insertions detected only by TEMP is significantly lower than insertions that were also detected by TIDAL or hemi-specific PCR (Welch’s *t*_27_ = 11.05, *P* = 1.33 × 10^−11^). (PNG 34 kb)

Additional file 5: Tables S3. PCR verification of insertions found by hemi-specific PCR. (XLSX 140 kb)

Additional file 6: Figure S2. PCR of annotated insertion sites. To verify the existence of some *P*-element insertions, we performed insertion-specific PCR. In the absence of a *P*-element insertion, the length of all PCR products was expected to be less than 500 bp. However, the main bands of PCR products at all locations were more than 500 bp, indicating the existence of insertion events. To determine if the insertions contain *P*-elements, we sequenced the PCR products (indicated by red rectangles). Sequencing results indicated the existence of *P*-element at each location. Notably, chr2L:20,917,521 a polymorphic insertion which is absent from the TEMP and TIDAL annotation. (PNG 35 kb)

Additional file 7: A shell script used to detect *P*-element insertions from hemi-specific PCR. (SH 2 kb)

Additional file 8: A Perl script used to pick up *P*-element derived read paired. (PL 2 kb)

Additional file 9: A Perl script used to pick up concordantly and uniquely mapping read pairs. (PL 997 bytes)

Additional file 10: A Perl script used to determine the breakpoints of *P*-element insertions. (PL 3 kb)

## Abbreviations

TE: Transposable element
TGS: Targeted genome re-sequencing
WGS: Whole genome re-sequencing
*X-TAS*: *X* chromosome telomere associated satellites
