# Supplementary figures and images for: Targeted identification of TE insertions in a Drosophila genome through hemi-specific PCR
Source: Mob DNA. 2017 Jul 28;8:10. doi: 10.1186/s13100-017-0092-1 (PMC5534036; doi:10.1186/s13100-017-0092-1)

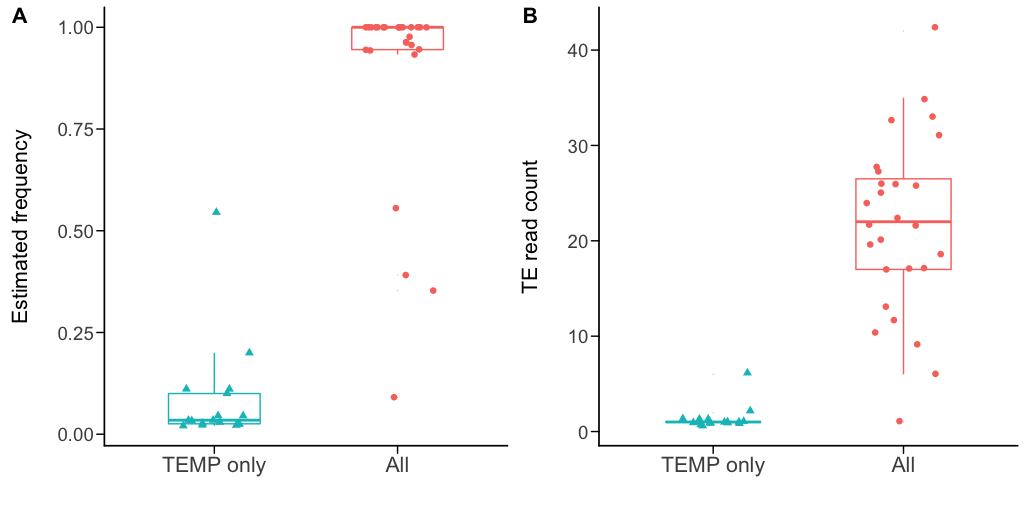

Supplement: Supplementary file 4 — The P-element insertions found only by TEMP have a low frequency (A) and are supported by few reads (B). P-element insertion detected by TEMP (two tail-to-tail insertions are excluded) are divided into two groups: the “TEMP only” group which contains insertions found only by TEMP, and the “All” group which contains insertions found by TEMP, TIDAL and hemi-specific PCR. Frequency (called penetrance by TEMP) was estimated from whole genome sequencing of the RAL-492 line. TE read count indicates the number of paired-end reads that support the inserted chromosome. Insertions annotated only by TEMP have a significantly lower estimated polymorphic frequencies than insertions also detected by TIDAL and hemi-specific PCR (Welch’s t 41 = 14.44, P < 2.2 × 10^−16^). In addition, the average number of reads supporting P-element insertions detected only by TEMP is significantly lower than insertions that were also detected by TIDAL or hemi-specific PCR (Welch’s t 27 = 11.05, P = 1.33 × 10^−11^). (PNG 34 kb) [file 13100_2017_92_MOESM4_ESM.png]

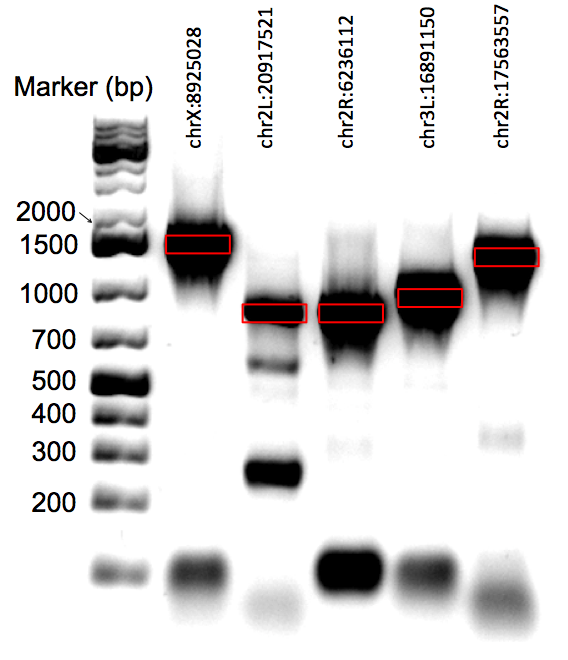

Supplement: Supplementary file 6 — PCR of annotated insertion sites. To verify the existence of some P-element insertions, we performed insertion-specific PCR. In the absence of a P-element insertion, the length of all PCR products was expected to be less than 500 bp. However, the main bands of PCR products at all locations were more than 500 bp, indicating the existence of insertion events. To determine if the insertions contain P-elements, we sequenced the PCR products (indicated by red rectangles). Sequencing results indicated the existence of P-element at each location. Notably, chr2L:20,917,521 a polymorphic insertion which is absent from the TEMP and TIDAL annotation. (PNG 35 kb) [file 13100_2017_92_MOESM6_ESM.png]
